# The Effects of Dietary Interventions on DNA Methylation: Implications for Obesity Management

**DOI:** 10.3390/ijms21228670

**Published:** 2020-11-17

**Authors:** Andrea Maugeri

**Affiliations:** Department of Medical and Surgical Sciences and Advanced Technologies “GF Ingrassia”, University of Catania, 95123 Catania, Italy; andrea.maugeri@unict.it

**Keywords:** diet, nutrition, epigenetics, weight loss

## Abstract

Previous evidence from in vivo and observational research suggested how dietary factors might affect DNA methylation signatures involved in obesity risk. However, findings from experimental studies are still scarce and, if present, not so clear. The current review summarizes studies investigating the effect of dietary interventions on DNA methylation in the general population and especially in people at risk for or with obesity. Overall, these studies suggest how dietary interventions may induce DNA methylation changes, which in turn are likely related to the risk of obesity and to different response to weight loss programs. These findings might explain the high interindividual variation in weight loss after a dietary intervention, with some people losing a lot of weight while others much less so. However, the interactions between genetic, epigenetic, environmental and lifestyle factors make the whole framework even more complex and further studies are needed to support the hypothesis of personalized interventions against obesity.

## 1. Introduction

Obesity is defined as excessive body fat deposition resulting in a disproportionate body weight for height [1]. This condition is usually associated with metabolic disorders, some type of cancer, and cardiovascular diseases [2], which account for a substantial burden for overweight and obese individuals [3]. In the past, the imbalance between caloric intake and energy expenditure was considered the main–and perhaps only–cause of excessive body fat accumulation. More recently, however, this simplistic view is gradually moving towards a more complex scenario involving environmental exposures, socioeconomic factors, and behaviors [4,5]. Among the latter, for instance, the quality of diet, level of physical activity, abuse of alcohol, and lack of sleep play a crucial role in maintaining an appropriate body weight [6,7,8,9,10,11,12,13]. As reported by the World Health Organization (WHO), more than 1.9 billion adults were overweight and of these 650 million were obese in 2016 [14]. These figures make overweight and obesity a priority for public health [3], raising the need for interventions aimed to tackle the progress of what can be considered a global epidemy [14]. Accordingly, several approaches and treatments have been proposed, such as dietary interventions, physical activity programs, drug administration, and bariatric surgery. 

In the past decades, several randomized controlled trials evaluated the effects of dietary interventions on body weight management and weight loss [15,16]. Although caloric restriction represents the easiest option to lose weight, improving the quality of diet can also be helpful [17]. However, there is high interindividual variation in weight loss after a dietary intervention, with some people losing a lot of weight while others much less so [15]. What makes this even more complex is that the interactions between genetic, epigenetic and the environmental factors might sustain important individual differences in body weight [18,19]. Genetic variants, for example, might explain important interindividual variation in the response to the same intervention [18,20], even if epidemiological research is needed to estimate the value of their contribution. In the same way, epigenetic mechanisms – including DNA methylation, histone modifications and noncoding RNAs – might play an important role in development of obesity from the early stages of life. Among these mechanisms, DNA methylation is one of the most extensively studied and best characterized. In mammals, DNA methylation is regulated by the activity of three DNA methyltransferases (DNMTs): while DNMT1 has a maintenance role, DNMT3a and 3b are de novo methylases. DNMT functions are associated with several key physiological processes, including genomic imprinting, X-chromosome inactivation, regulation of gene expression, maintenance of chromosome integrity through chromatin modulation, DNA stabilization and DNA-protein interactions [21]. Aberrant DNMT expression and activity are involved in several diseases including cardiovascular diseases, obesity, type-2 diabetes and cancer [22,23,24]. Mounting evidence from observational research has suggested a role in the DNA methylation process of nutrients and foods involved in one-carbon metabolism, as well as that of healthy dietary patterns [25]. In line, some experimental studies investigated the effect of dietary interventions, for example based on folate supplementation and adherence to the Mediterranean diet. In 2018, ElGendy and colleagues summarized experimental studies investigating the effects of dietary interventions on DNA methylation [26]. Specifically, the authors indicated that supplementation with folic acid - an important methyl donor in the DNA methylation process - differently but markedly affects DNA methylation levels in blood samples. Differences, however, depended on study population, sample type, and DNA methylation signature analyzed [26]. ElGendy and colleagues also described early results from experimental studies on the effect of weight-loss programs on DNA methylation signatures [26]. In fact, changes in DNA methylation might be involved in predisposition to obesity and in different response to dietary interventions. It has been already suggested that epigenomic programming of metabolism during the prenatal period – also known as metabolic imprinting – might affect the risk of obesity and other disorders over the lifetime [27,28]. Excessive maternal gestational weight during pregnancy, for example, is a risk factor for developing obesity at birth, as well as during infancy and adolescence [29]. Given that, birthweight can also be considered as a useful surrogate marker of fetal nutrition with a dual effect on the risk of obesity in the later phases of life: in fact, previous studies associated both high and low birthweight to the risk of obesity, excessive body fat, and metabolic disorders [30,31,32,33]. However, how much this transgenerational effect depends on epigenetics still remains to be elucidated [34,35,36]. 

**Figure 1:**
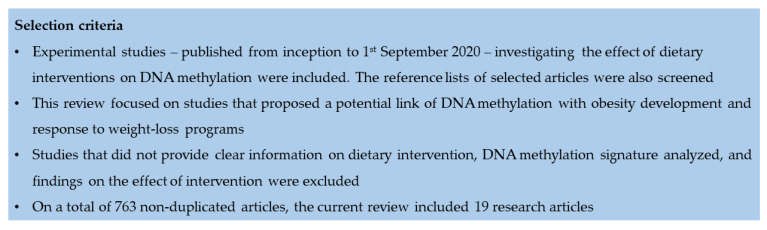
Literature search and selection criteria for experimental studies examining the effects of dietary interventions on DNA methylation.

Here, I first described recent findings on the potential relationship between dietary interventions during pregnancy and DNA methylation in cord blood of newborns. Next, I collected experimental studies investigating whether DNA methylation changes - following dietary interventions in adults - might vary according to their birthweight. Finally, I summarized evidence on the effects of weight-loss programs on DNA methylation signatures, taking into account their potential relationship with response to treatment. To do that, a literature search was carried out on PubMed and Web of Science databases by using the MESH terms “Diet” and “DNA Methylation”. Inclusion and exclusion criteria used for study selection are reported in the Figure 1, while methodological characteristics of included studies are summarized in Table 1.

## 2. Dietary Interventions during Pregnancy

Potential preventive strategies against obesity should start as early as possible, even during the perinatal period [1]. A recent review of observational studies on mother-child pairs summarized how the interaction between dietary factors and DNA methylation might be related to pregnancy outcomes [25]. Interestingly, the main diet-associated changes in DNA methylation regarded genes in the metabolic and growth pathways, such as insulin-like growth factor 2 (IGF2). This gene encodes for a protein hormone with growth-regulating, insulin-like and mitogenic activities, especially during pregnancy [25]. 

In spite of promising findings from observational studies, evidence from experimental research is still scarce. To my knowledge, the study by Lee and colleagues was the first investigating the effect of dietary interventions during pregnancy on DNA methylation in newborns [37]. The intervention consisted in dietary supplementation with ω-3 polyunsaturated fatty acid (PUFA) at 18–22 weeks of gestation. The authors reported an association between ω-3 PUFA supplementation and long interspersed nucleotide elements 1 (LINE-1) methylation levels, especially among newborns of smoker women. It is worth mentioning that observational research associated LINE-1 methylation with several disease in adulthood, including cancer, neurodegenerative diseases, obesity, and metabolic disorders [49,55,56,57,58,59,60,61,62,63,64]. In 2018, Geraghty and colleagues evaluated the effect of an intervention based on dietetic consulting and written resources to promote healthy dietary habits in general, and low glycemic index diet in particular [38]. Specifically, women in the intervention group were recommended to follow an eucaloric diet but replacing high glycemic foods with low glycemic alternatives. In the discovery cohort of 60 mother-child pairs, children born from mothers in the intervention group exhibited high variation in DNA methylation, especially in genes related to cardiac and immune functions. These results, however, were inconsistent with those obtained in the replication cohort, and no associations with maternal body mass index (BMI), infant sex, or birthweight were evident [38]. 

## 3. The Effect of Interventions in Adults According to Their Birthweight

With this in mind, a peculiar study design has been adopted to compare the effect of dietary interventions on DNA methylation between Danish men with normal or low birthweight [39]. Both groups were subjected to a control diet followed by a five-day high-fat overfeeding diet or vice versa, while skeletal muscle biopsies were collected to measure methylation of proliferator-activated receptor-γ, coactivator-1α (PPARGC1A) gene. During the control diet, PPARGC1A methylation was markedly higher in low birthweight individuals than in their counterpart. However, after the overfeeding diet, its methylation level increased only in normal birthweight men [39]. The same research group then evaluated PPARGC1A methylation level in the subcutaneous adipose tissue but achieving opposite findings [40]. Indeed, the high-fat overfeeding intervention increased PPARGC1A methylation level in low birthweight but not in normal birthweight individuals [40]. Nevertheless, these results were important since they suggested that dietary interventions might differently act depending on tissues. It is worth mentioning that PPARGC1A encodes for an important transcriptional coactivator involved in mitochondrial biogenesis and oxidative phosphorylation [65,66]. For this reason, PPARGC1A is highly expressed in tissues with high energy demand (e.g. skeletal muscle) [65] and lowly expressed in other tissues, such as white adipose tissue and pancreas [67]. In particular, decreased expression of PPARGC1A might cause insulin resistance by influencing several cellular functions (i.e. mitochondrial function, lipid oxidation, microvascular flow, and oxidative stress) [68,69,70]. However, it could play different roles in skeletal muscle and subcutaneous adipose tissue. To deeply understand how high-fat overfeeding affected DNA methylation in skeletal muscle, the authors also conducted two separate genomewide methylation studies [41,42]. In the first one, no significant differences in DNA methylation were evident between low and normal birthweight individuals. Yet, the overfeeding diet produced more DNA methylation changes in normal birthweight individuals than in those with low birthweight [42]. According to their results, the authors speculated that the decreased plasticity observed in low birthweight individuals might interfere with protective functions of various pathways (e.g. inflammation) and thus might increase their risk for insulin resistance and type 2 diabetes [42]. In the second genomewide study, the authors evaluated the effect of a three-day weight-maintaining diet followed by a five-day high-fat overfeeding diet only in men with normal birthweight [41]. Interestingly, the intervention was associated with more than 6500 differentially methylated regions and only a part of these changes reversed after two months from the intervention. Further analysis underlined that the majority of these differentially methylated regions were associated with pathways involved in inflammation, reproductive activities and cancer [41].

A similar but more complex approach has been adopted to uncover how overfeeding diet affected DNA methylation signatures in subcutaneous adipose tissue [43]. In 2016, the study by Gillberg and colleagues included a discovery cohort (made of low-birthweight men and BMI-matched control men with normal birthweight) and two replication cohorts (composed of elderly monozygotic and dizygotic twins and healthy young individuals, respectively) [43]. In the discovery cohort, there were 53 differentially methylated regions associated with birthweight, such as loci within Fatty Acid Desaturase 2 (FADS2) and Complexin 1 (CPLX1) genes [43], which in turn have been associated with type 2 diabetes [71] and glucose-stimulated insulin release [72], respectively. In the replication cohorts, instead, the intervention was linked to 652 differentially methylated regions within genes that were predominantly related to metabolic pathways (e.g. insulin-like growth factor-binding protein 5, IGFBP5; Solute Carrier Family 2 Member 4, SLC2A4) [43]. More recently, Hjort and colleagues compared the effects of a 72 h control diet of precooked meals followed by 36 h of fasting between men with normal and low birthweight [44]. They showed that leptin (LEP) and adiponectin (ADIPOQ) methylation levels were higher in subcutaneous adipose tissue of low birthweight subjects than in their normal birthweight counterpart. Interestingly, 36 h fasting was associated with increasing DNA methylation levels only in normal birthweight individuals. It is worth mentioning that LEP and ADIPOQ are among the most important adipokines correlated with adipose tissue mass, visceral adiposity, and body fat percentage [73,74].

## 4. Dietary Interventions against Obesity and Related Disorders 

In the last decade, the research on dietary risk factors for obesity also aimed to understand diet-associated changes in DNA methylation. In the context of experimental research, studying DNA methylation could be helpful to uncover molecular mechanisms underpinning different response to weigh loss programs and interventions. In 2011, Milagro and colleagues analyzed the genomewide methylation profile in blood samples of 25 overweight or obese men subjected to an energy-restricted program [22]. The intervention consisted of an eight-week energy-restricted diet with 53% of energy from carbohydrates, 30% from fats, and 17% from proteins. Prior to intervention, ATPase Phospholipid Transporting 10A (ATP10A) and CD44 methylation levels differed between responders and nonresponders. After eight weeks of intervention, instead, the response was associated with methylation status of Wilms’ tumor 1 (WT1) gene [22]. These findings were partially confirmed by Samblas and colleagues in 2018. In fact, the authors demonstrated that CD44 methylation level was lower in individuals who responded less to a weight-loss program if compared with high responders [45]. Interestingly, ATP10A encodes an amino-phospholipid translocase related to lipid trafficking and maintenance of the plasma membrane, which is probably involved in modulating body fat [75]. For this reason, the depletion of ATP10A was used to induce clinical traits of obesity in mice [76]. CD44, instead, is a cell-surface glycoprotein indirectly involved in inflammation and fibrosis [77]. For instance, CD44 expression was upregulated in the liver of obese patients with steatohepatitis, while it was downregulated in subcutaneous adipose tissue after weight loss [78]. WT1 encodes for Kruppel-like zinc-finger protein that can act as oncosuppressor or oncogene in relation to cell type [79]. In line, methylation status of WT1 was extensively investigated in cancer research, but much less in obese patients. This encourage further studies investigating the molecular basis underlying the association observed by Milagro and colleagues. In the same year, Cordero and colleagues analyzed Leptin and Tumor Necrosis Factor Alpha (TNF-α) methylation levels in adipose tissue of 27 obese women subjected to a similar eight-week energy-restricted diet [46]. Interestingly, better response to the intervention was associated with lower Leptin and TNF-α methylation. Previous studies already demonstrated that the proinflammatory cytokine TNF-α was overexpressed in obesity and related metabolic disorders and that its production was mediated by epigenetic modifications [80]. Moreover, methylation status of TNF-α in blood samples was previously associated with the response to a low-calorie diet [47]. 

In 2015, Nicoletti and colleagues recruited 45 women grouped as follows: 22 obese women subjected to a six-month energy restriction program; 14 obese women subjected to a hypocaloric diet followed by bariatric surgery; nine normal weight women [48]. According to this classification, the authors compared IL-6, Serpin Family E Member 1 (SERPINE-1), and LINE-1 methylation level in blood samples. In particular, SERPINE-1 and LINE-1 methylation levels were similar between different groups and not associated with obesity traits. However, LINE-1 methylation was associated with serum glucose levels prior to interventions. Nicoletti and colleagues also noted that IL-6 methylation level was higher in obese women subjected to energy restriction and lower in those who underwent to bariatric surgery [48]. In general, obesity is associated with the release of inflammatory factors - such as IL6 - which in turn induce a chronic state of low-grade inflammation [81,82]. On the contrary, weight loss programs might reduce the inflammatory state of obese patients [83,84]. This evidence partially supports the increased IL6 methylation level observed in the energy-restricted group [48], which may be associated with lower gene expression and protein level. The opposite effect observed in the bariatric surgery group, instead, should be confirmed by future studies. Although the authors speculated that both surgical procedure and fast weight loss could promote an inflammatory state [48], further research is needed to support this hypothesis. 

Results on LINE-1 methylation reported by Nicoletti and colleagues were in line with those from observational studies [85,86,87,88,89,90,91,92]. Accordingly, Delgado-Cruzata and colleagues treated 24 overweight and sedentary female breast cancer survivors with a six-month weight loss program. The interventions aimed to increase physical activity and to reduce caloric intake in general and from fats in particular. The authors showed that LINE-1 methylation in blood samples significantly increased after 6–12 months from the intervention, and that its level was positively associated with changes in percentage of body fat and fasting glucose concentration [49]. These findings were consistent with those obtained by researcher of the Metabolic Syndrome Reduction in Navarra (RESMENA) project [50]. In this study, the intervention group consisted of adults subjected to seven meals per day with a macronutrient distribution of 40% total caloric value from carbohydrates, 30% from proteins and 30% from lipids. Subjects in the control group, instead, were asked to adhere to the American Heart Association (AHA) guidelines (i.e. three to five meals per day and a macronutrient distribution of 55% total caloric value from carbohydrates, 15% from proteins, and 30% from lipids). Interestingly, baseline LINE-1 methylation level was higher in patients with higher weight loss independent of intervention [50]. Yet, other studies found opposite or inconsistent results about the putative association between dietary interventions and LINE-1 methylation. For instance, Martin-Nunez and colleagues showed a reduction in LINE-1 methylation level after a 12-month intervention aiming to promote adherence to the Mediterranean diet and exercise [51]. Duggan and colleagues, instead, failed in demonstrating any significant difference in LINE-1 methylation level among overweight women randomized to three different intervention groups [52].

Finally, there were other studies that needed further confirmation due to their specific nature. This was the case, for example, of the study by Samblas and colleagues in 2016. The author, indeed, investigated the effect of dietary interventions on DNA methylation of genes involved in the circadian clock system [53]. The intervention consisted in the promotion of Mediterranean diet and physical activity among 61 overweight or obese women. This research, for the first time, pointed out an association between BMAL1 (brain and muscle aryl hydrocarbon receptor nuclear translocator like protein 1) methylation and an intervention, which in turn resulted in weight loss and decreased blood lipids levels. BMAL1 is a transcription factor that regulates the mammalian clock machinery. Interestingly, previous studies already demonstrated the involvement of BMAL1 in the regulation of adipogenesis and lipid metabolism, as well as in hyperlipidemic and hyperglycemic periods in obese subjects [53]. Another peculiar study was that by Sun and colleagues, which tested the potential interactions between dietary interventions and NFATC2IP (Nuclear Factor Of Activated T Cells 2 Interacting Protein) genotype, DNA methylation, and gene expression on two-year weight change [54]. Dietary interventions consisted of four low-calorie diets with different macronutrient composition. Interestingly, NFATC2IP methylation exhibited an opposite effect on weight-loss in response to high-fat or low-fat diets. Moreover, NFATC2IP methylation mediated about half of the effect of rs11150675 NFATC2IP genetic polymorphism on two-year weight-loss following the high-fat diet [54]. To my knowledge, this is the first evidence of an interaction between genetic variants and DNA methylation on response to weight-loss programs, and hence further studies should be encouraged to corroborate these findings. 

## 5. Discussion

**Figure 2:**
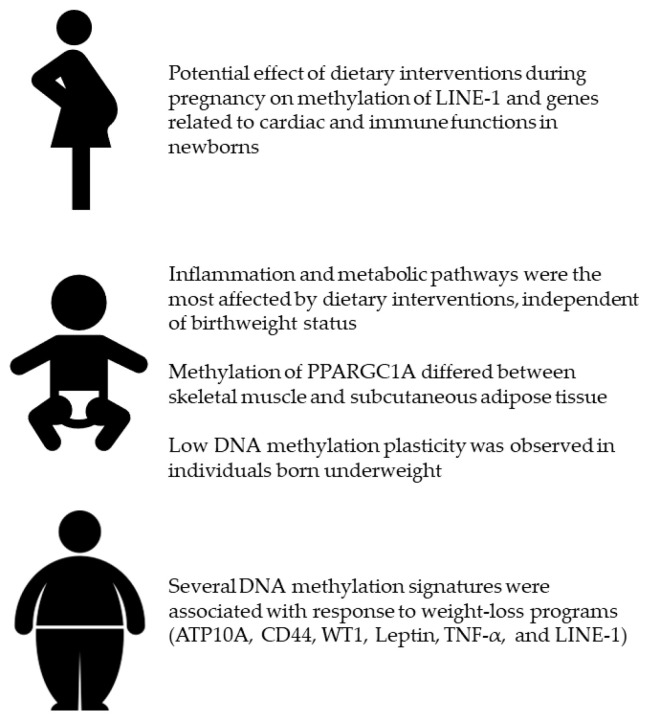
Effect of dietary interventions during pregnancy, in adults according to their birthweight, and in overweight or obese individuals.

Several lines of evidence already described the relationship of dietary factors (i.e. nutrients, foods, and dietary patterns) with DNA methylation signatures that might be involved in health and diseases [25]. Although the majority of findings originated from in vivo and observational research, ElGendy and colleagues summarized experimental studies conducted to elucidate the effect of various dietary interventions [26]. Their compelling work demonstrated how different interventions differentially affected DNA methylation signatures in blood samples and other specimens. For instance, a lot of studies conducted among “healthy” people evaluated the effect of folate supplementation [93,94] and Mediterranean diet promotion [95,96]. Interestingly, folate intake and supplementation differently affected DNA methylation signatures, and this difference was due to participants’ characteristics, sample types and genomic sites under investigation [93,94]. With respect to the Mediterranean diet, the PREDIMED study produced the most interesting results, suggesting that the majority of differentially methylated regions were located in genes involved in inflammation, immunocompetence, signal transduction and metabolic pathways [95,96]. ElGendy and colleagues also reported that some DNA methylation changes might be associated with obesity development and response to weight-loss program [26]. For this reason, the present review collected all the experimental studies investigating the effect of dietary interventions on DNA methylation, with a particular focus on those suggesting a potential link with obesity. Specifically, I focused on (i) dietary interventions during pregnancy (i.e. a crucial period for developing obesity later in life); (ii) studies evaluating if birthweight might affect DNA methylation changes following a dietary intervention; (iii) and those investigating the effect of weight-loss and/or energy-restricted programs. Figure 2 illustrates the most important findings presented in the current review. 

With regard to the first point, however, experimental studies on mother-child pairs were scarce, with some controversial results that therefore required further investigation [38]. Yet, birthweight is one of the main neonatal outcomes associated with the risk of obesity and related disorders in the childhood, adolescence, and adulthood [97]. For this reason, several studies compared the effect of dietary interventions on DNA methylation between individuals born underweight or normal weight [39,40,41,42]. The main purpose of these studies was to provide an explanation of differences in the response to different dietary interventions when patients were stratified by their birthweight. These studies – conducted with a similar study design – indicated several DNA methylation signatures that differed between individuals with normal or low birthweight [39,40,41,42]. In this framework, methylation of PPARGC1A was that has attracted more interest due to its opposite path in skeletal muscle and subcutaneous adipose tissue [39,40]. However, tissue-specific effects of PPARGC1A methylation still remain to be elucidated. Another hypothesis to test regards the low DNA methylation plasticity observed in individuals born underweight. Indeed, subjects with low birthweight exhibited a less marked effect of dietary intervention on their DNA methylation profile [42]. In fact, DNA methylation changes-induced by dietary interventions in individuals with normal birthweight-might stimulate some pathways (e.g. inflammation) with protective functions against obesity-related disorders [42]. By contrast, the low plasticity observed in those with low birthweight might increase their risk for insulin resistance and type 2 diabetes [42]. Of note, inflammation and metabolic pathways seemed the most affected by dietary interventions independent of birthweight status [39,40,41,42]. Overall, these findings provide grounds to hypothesize that dietary interventions might modulate the DNA methylation processes, and that their effects are likely related to the risk of obesity and to different response to weight loss programs. In line, some studies evaluated how dietary interventions based on energy restriction might induce changes in DNA methylation. Interestingly, several DNA methylation signatures seemed associated with weight-loss response (e.g. ATP10A, CD44, WT1, Leptin, TNF-α, and LINE-1) [22,46,48,49,50]. However, the current review also raised several aspects that might prevent the comparison between different studies. Several studies were not randomized, while others did not report clearly report some important methodological aspects. This was important because differences in study design and dietary interventions, peculiar characteristics of the study population, as well as heterogeneity in sample types, loci analyzed, and methods used for estimating DNA methylation, might lead to different - and sometimes opposing - results. 

In conclusion, findings described in the present review are promising, suggesting the possibility to individualize the weight-loss interventions according to specific DNA methylation signatures. However, further studies conducted on large-size populations with a standardized protocol are necessary to produce robust evidence and to integrate DNA methylation data with genetic profile and other characteristics of patients. 

## Figures and Tables

**Table 1:** Summary of experimental studies examining the effects of dietary interventions on DNA methylation.

First Author and Year of Publication	Country	Study Design	Study Population	Age (years)	Dietary Intervention	DNA Methylation Marker	Method	Samples
Lee 2013 [37]	Mexico	RCT	Pregnant women at 18–22 weeks of gestation	18–35	Daily supplementation of 400 mg DHA or a placebo	LINE-1, IFNγ-1, IFNγ-2, TNF-alpha, GATA3, IL10, IL13, STAT3, FOXP3	Pyrosequencing	Cord blood
Geraghty 2018 [38]	Ireland	RCT	The discovery cohort included 60 sex-matched mother-child pairs (30 participants in the intervention arm and 30 participants in the control arm). The replication cohort consisted of different 60 sex-matched mother-child pairs	Mean = 32.8 (SD = 4.5) in the intervention group; 33.9 (SD = 4.2) in the control group	Eucaloric diet but replacing high glycemic index foods with low glycemic index alternatives	Genome-wide methylation profile	Illumina Infinium MethylationEPIC BeadChip Array and Sequenom MassARRAY	Cord blood
Brøns 2010 [39]	Denmark	Randomized crossover study	20 low birthweight men and 26 normal birthweight controls	Mean = 24.6 (SD = 1) and 24.2 (SD = 0.5) in normal birthweight and low birthweight, respectively	Five-day high-fat overfeeding diet and a control diet in a randomized order separated by six to eight weeks	PPARGC1A	Bisulfite sequencing	Skeletal muscle biopsy
Gillberg 2014 [40]	Denmark	Randomized crossover study	19 low birthweight men and 26 normal birthweight controls	23–27	Five-day high-fat overfeeding diet and a control diet in a randomized order separated by six to eight weeks	PPARGC1A	Bisulfite sequencing	Subcutaneous adipose tissue
Jacobsen 2012 [41]	Denmark	Randomized crossover study	25 young men with normal birthweight	Mean = 24.6 (SD = 1.1)	Three-day weight-maintaining diet followed by five-day high-fat overfeeding diet	Genomewide methylation profile	Infinium HumanMethylation27K bead chip, Sequenom’s MassARRAY EpiTYPER and pyrosequencing	Skeletal muscle biopsy
Jacobsen 2014 [42]	Denmark	Randomized crossover study	17 low birthweight men and 23 normal birthweight controls	Mean = 24.6 (SD = 1) and 24.2 (SD = 0.5) in normal birthweight and low birthweight, respectively	Five-day high-fat overfeeding diet and a control diet in a randomized order separated by six to eight weeks	Genomewide methylation profile	Infinium HumanMethylation27K bead chip	Skeletal muscle biopsy
Gillberg 2016 [43]	Denmark	Randomized crossover study	The discovery cohort included 16 normal birthweight men and 24 age- and BMI-matched control men with normal birthweight. Two replication cohorts consisted of 142 elderly monozygotic and dizygotic twins and 17 healthy young individuals, respectively	NA	Three-day weight-maintaining diet followed by 5-day high-fat overfeeding diet	Genomewide methylation profile	Infinium HumanMethylation450K bead chip	Subcutaneous adipose tissue
Hjort 2017 [44]	Denmark	Non-RCT	21 low birthweight men and 18 normal birthweight controls	Mean = 24.6 (SD = 1.2) and 24.8 (SD = 1.4) in normal birthweight and low birthweight, respectively	72 h control diet of precooked meals followed by 36 h of fasting with ad libitum water	LEP and ADIPOQ	Sequenom MassARRAY	Subcutaneous adipose tissue
Milagro 2011 [22]	Spain	Non-RCT	25 overweight or obese men	NA	Eight-week energy-restricted diet with 53% of energy from carbohydrates, 17% from proteins and 30% from fats	Genomewide methylation profile	Infinium HumanMethylation27K bead chip and MALDI-TOF mass spectrometry	Blood
Samblas 2018 [45]	Spain	RCT	47 adults with metabolic syndrome randomized to an energy-restricted dietary intervention	NA	Seven meals per day with a macronutrient distribution of 40% total caloric value from carbohydrates, 30% from proteins and 30% from lipids	Genomewide methylation profile	Infinium HumanMethylation27K bead chip and Sequenom’s MassARRAY EpiTYPER	Blood
Cordero 2011 [46]	Spain	Non-RCT	27 obese women	32–50	Eight-week energy-restricted diet with 55% of energy from carbohydrates, 15% from proteins and 30% from fats	Leptin and TNF-alpha	Methylation-specific PCR	Adipose tissue
Campion 2009 [47]	Spain	Non-RCT	24 obese individuals	Mean = 34 (SD = 4)	Eight-week energy-restricted diet	TNF-alpha	Bisulfite sequencing	Blood
Nicoletti 2015 [48]	Brazil and Spain	Non-RCT	45 women randomized to three different intervention groups	Mean = 31.7 (SD = 8.6) in the control group; 52.6 (SD = 9.9) in the energy restriction group; 35.5 (SD = 10.1) in the bariatric surgery group	Six-month energy restriction program; hypocaloric dietary treatment followed by bariatric surgery; control group	LINE-1, IL-6, and SERPINE-1	Methylation-sensitive high-resolution melting analysis	Blood
Delgado-Cruzata 2015 [49]	USA	Non-RCT	24 overweight and sedentary female breast cancer survivors	Mean = 52.2 (SD = 8.7)	Six-month weight loss program aimed to increase physical activity to 90 minutes per week, to reduce caloric intake, and to distribute caloric intake as 45% from protein, 30% from carbohydrates, and 25% from fats	Global DNA methylation and LINE-1	LUMA, pyrosequencing and MethyLight assay	Blood
Garcia-Lacarte 2016 [50]	Spain	RCT	96 adults with metabolic syndrome randomized to two energy-restricted dietary interventions	NA	Seven meals per day with a macronutrient distribution of 40% total caloric value from carbohydrates, 30% from proteins and 30% from lipids; the control group followed the American Heart Association guidelines	LINE-1	Methylation-sensitive high-resolution melting analysis	Blood
Martin-Nunez 2014 [51]	Spain	Non-RCT	310 obese participants	Mean = 53.6 and 54.6 in the control and intervention group, respectively	12-month intervention program based on the promotion of Mediterranean diet and exercise	LINE-1 and SCD1	Pyrosequencing	Blood
Duggan 2014 [52]	USA	RCT	298 overweight women randomized to four groups	Mean = 57.9 (SD = 4.9)	A control group and 12-month interventions aimed to reduce energy intake, to increase physical activity, or both	LINE-1	Pyrosequencing	Blood
Samblas 2016 [53]	Spain	Non-RCT	61 overweight or obese women	Mean = 42.2 (SD = 11.4)	Nine-month program to promote Mediterranean diet, physical activity, nutritional and behavioral education	BMAL1, NR1D1, and CLOCK	Sequenom’s MassARRAY EpiTyper	Blood
Sun 2018 [54]	USA	RCT	692 individuals randomized to four energy-reduced diets varying in macronutrients	Mean = 51.4	Two-year interventions, consisting of two high-fat diets low in carbohydrate and two low-fat diets high in carbohydrate diets	Genomewide methylation profile	Infinium HumanMethylation450 BeadChip	Blood

## Abbreviations

WHO	World Health Organization
DNMTs	DNA methyltransferases
IGF2	Insulin-like growth factor 2
LINE-1	Long interspersed nuclear elements 1
BMI	Body Mass Index
PPARGC1A	Proliferator-activated receptor-γ, coactivator-1α
FADS2	Fatty Acid Desaturase 2
CPLX1	Complexin 1
IGFBP5	Insulin-like growth factor-binding protein 5
SLC2A4	Solute Carrier Family 2 Member 4
LEP	Leptin
ADIPQ	Adiponectin
MTHFR	Methylenetetrahydrofolate reductase
PREDIMED	Prevención con Dieta Mediterránea
GOLDN	Genetics of Lipid Lowering Drugs and Diet Network
LPP	Lipoma-preferred partner
APOA5	Apolipoprotein A-5
SREBF1	Sterol regulatory element-binding transcription factor 1
ABCG1	ATP-binding cassette sub-family G member 1
CPT1A	Carnitine palmitoyl-transferase 1-A
PUFA	Polyunsaturated fatty acids
SFA	Saturated fatty acids
FTO	Alpha-ketoglutarate dependent dioxygenase
IL6	Interleukin 6
INSR	Insulin receptor
NEGR1	Neuronal growth regulator 1
POMC	Proopiomelanocortin
ATP10A	ATPase Phospholipid Transporting 10A
WT1	Wilms’ tumor 1
TNF-α	Tumor Necrosis Factor Alpha
SERPINE-1	Serpin Family E Member 1
RESMENA	Metabolic Syndrome Reduction in Navarra
AHA	American Heart Association
BMAL1	Brain and muscle aryl hydrocarbon receptor nuclear translocator–like protein 1
NFATC2IP	Nuclear Factor Of Activated T Cells 2 Interacting Protein
